# Beam heating from a fourth-generation synchrotron source

**DOI:** 10.1107/S160057752100669X

**Published:** 2021-07-20

**Authors:** Eleanor Lawrence Bright, Carlotta Giacobbe, Jonathan P. Wright

**Affiliations:** a European Synchrotron Radiation Facility, 71 Avenue des Martyrs, 38040 Grenoble, France

**Keywords:** beam heating, radiation damage

## Abstract

The high levels of flux available at a fourth-generation synchrotron are shown to have significant beam heating effects for high-energy X-rays and radiation hard samples, leading to temperature increases of over 400 K with a monochromatic beam. These effects have been investigated at the ID11 beamline at the recently upgraded ESRF Extremely Brilliant Source, using thermal lattice expansion to perform *in situ* measurements of beam heating. Results showed significant increases in temperature for metal and ceria samples, which are compared with a lumped thermodynamic model, providing a tool for estimating beam heating effects. These temperature increases may have a drastic effect on samples and measurements, such as the rapid recrystallization of a copper wire shown here. These results demonstrate the importance of beam heating and provide information needed to consider, predict and mitigate these effects.

## Abstract 2

The high levels of flux available at a fourth-generation synchrotron are shown to have significant beam heating effects for high-energy X-rays and hard condensed matter samples, leading to temperature increases of over 400 K with a monochromatic beam.

## 1. Introduction   

Fourth-generation synchrotron X-ray sources bring increasing levels of flux and coherence, allowing unprecedented levels of resolution for a wide range of techniques, but with increasing risk of radiation damage. The high flux achievable at synchrotrons has been well known to cause damage in biological samples at around 5–20 keV (Ravelli & Garman, 2006 ▸; Garman, 2010 ▸; Garman & Weik, 2019 ▸); however, with increasing flux we have found that radiation effects become significant even for metal samples and high-energy X-rays through beam heating. With the Extremely Brilliant Source upgrade at the European Synchrotron Radiation Facility (ESRF-EBS), we show that a monochromatic 43.44 keV beam can cause temperature increases as large as 400 K. These significant increases in sample temperature may change sample properties such as lattice parameters and drive significant chemical or physical changes. Properties under investigation may be temperature dependent for many materials science experiments, such as mechanical response, chemical behaviour, microstructure *etc*., making it vital that any changes in temperature are known. If the materials being measured are used as standards, temperature can play a significant role in the accuracy of measurements. These factors make X-ray beam heating a potential issue for all samples.

While beam heating and radiation effects on biological samples are usually overcome with cryo-cooling, high-speed measurements, helical scans, or a combination (Holton, 2009 ▸; Stern *et al.*, 2009 ▸; Borek *et al.*, 2010 ▸), these measures may not be appropriate for many materials science experiments. Consequently, we need to be able to understand and model beam heating to predict the severity of the problem and determine possible methods of mitigation. Past studies have investigated beam heating effects on samples, modelling the effects of sample geometry, time, thermal conductivity, heat sinks and other parameters (Helliwell, 1984 ▸; Kuzay *et al.*, 2001 ▸; Kriminski *et al.*, 2003 ▸; Hopkins & Thorne, 2016 ▸; Wallander & Wallentin, 2017 ▸; Bonino *et al.*, 2020 ▸). Some experimental investigations have shown increases in temperature in a third-generation synchrotron beam (Snell *et al.*, 2007 ▸; Rosenthal *et al.*, 2014 ▸; Warren *et al.*, 2019 ▸); however, these studies mostly consider only the implications for soft, biological samples and lack a clear comparison between model and experiment.

Here, we provide experimental investigation into these effects at a fourth-generation synchrotron by measuring sample temperature changes via thermal lattice expansion using *in situ* X-ray diffraction performed at the ESRF-EBS. By designing samples to maximize effects and simplify the thermodynamics of the system, we set up a quantitative comparison to a model for beam heating to help to predict and understand the severity of the problem.

## 2. Experiment   

Experiments were performed at the Materials Science Beamline (ID11) at the ESRF. The recent Extremely Brilliant Source (EBS) upgrade at the ESRF has increased the brilliance of the X-ray beams produced by many of the beamlines compared with the previous source (Raimondi, 2016 ▸). With this upgrade, ID11 can now achieve a flux of up to 2 × 10^13^ photons s^−1^ at an energy of 43.44 keV and bandpass of 10^−3^ using two in-vacuum undulators and a double bent crystal monochromator operating in horizontal Laue geometry (Wright *et al.*, 2020 ▸).

**Figure 1:**
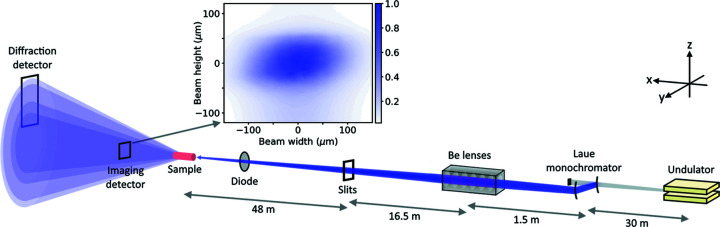
Schematic of the experimental set-up at the ID11 beamline, with a plot showing an image of the focused beam.

Measurements were performed at 96 m from the source, as shown in Fig. 1 ▸, with far-field diffraction and near-field imaging set-up for *in situ* measurements. FReLoN cameras were used for collecting imaging and diffraction data, with imaging used to determine sample dimensions and alignment relative to the beam. Diffraction data were used to determine sample temperature using lattice expansion. The experimental hutch was maintained at a stable temperature, keeping the sample environment at 294 K.

Flux on the samples was maximized by using a beryllium in-vacuum compound refractive lens transfocator that is 31.5 m from the source to give 1:2 focusing of the beam onto the sample (Vaughan *et al.*, 2011 ▸). The profile of the focused beam is displayed in Fig. 1 ▸, where it can be seen that the beam has an FWHM of 80 µm × 140 µm. Time-resolved instantaneous heating measurements were performed by acquiring data every 0.01 s when the focusing lenses were inserted into the beam. Flux-dependent measurements were performed by opening upstream slits that were close to the lenses while collecting diffraction data. The incident flux values were determined using a silicon diode placed in the beam before the sample and conversion to photons s^−1^ via the method provided by Owen *et al.* (2009 ▸).

Beam heating measurements were performed on samples with different absorption coefficients, sample geometry, and orientation with respect to the beam. Aluminium wire samples with a diameter of 0.5 mm were chosen as an example with a low attenuation coefficient, μ, while 0.19 and 0.025 mm-diameter copper wires provide a higher-μ example. As the 0.19 mm-diameter wire is similar to the focused beam size, these copper samples were designed to give large increases in temperature. A 0.5 mm capillary of CeO_2_ powder, commonly used for calibration in X-ray diffraction measurements, was also investigated. Calibration of sample–detector distance was done using CeO_2_ at low flux. This CeO_2_ sample was then also measured at high flux.

Wire samples were mounted with their length oriented along either *x*, the direction of the X-ray beam, or *y*, the horizontal direction of X-ray polarization. To approximate a lumped model system, samples should be thermally isolated. To achieve this, wire samples were mounted end-on to a small strip of Kapton tape. This is not as stable as mechanical mounting; when the sample is in the orientation for achieving the highest increase in temperature, any movement from this position will decrease the temperature.

## 3. Results from example samples   

**Figure 2:**
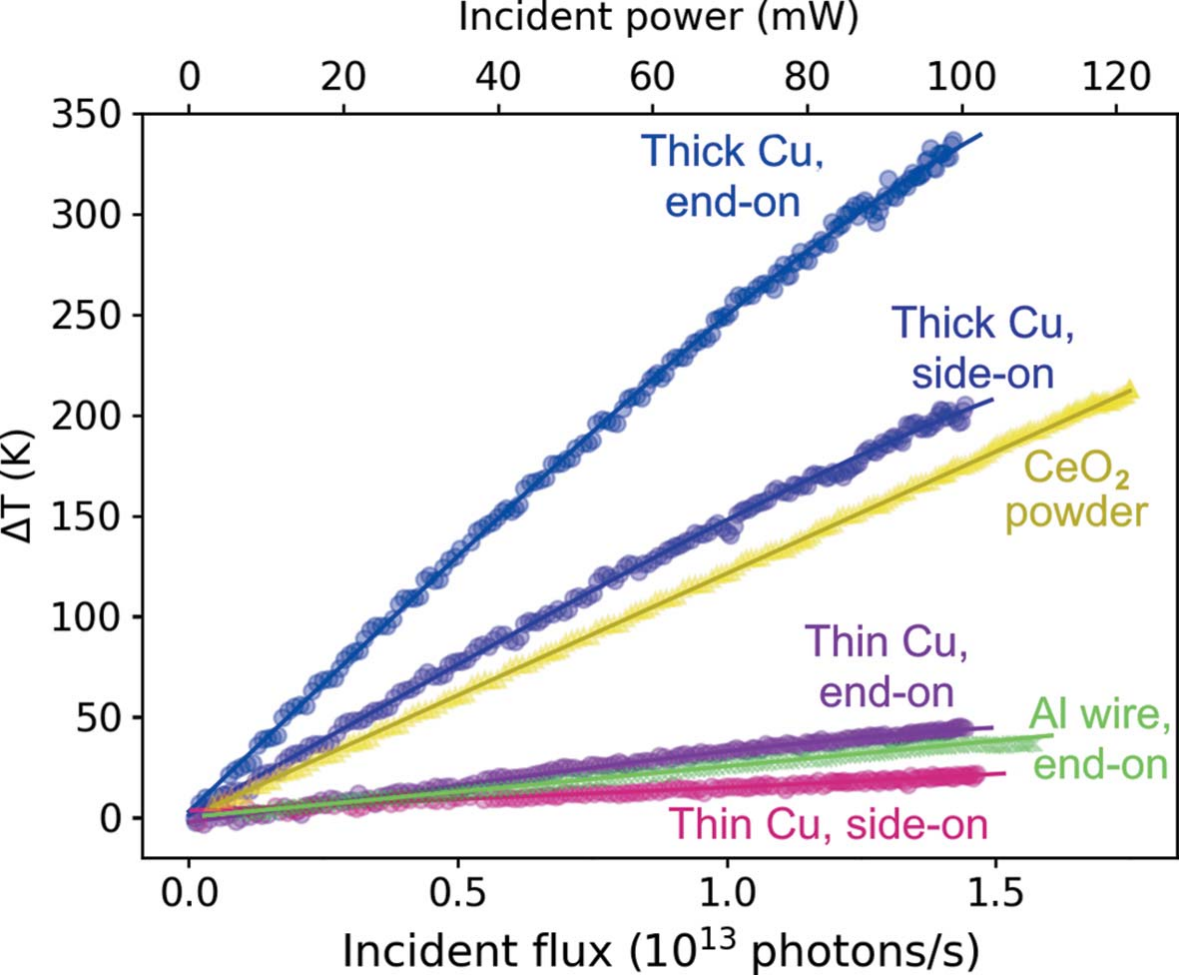
Change in temperature of samples as a function of incident flux, with 0.19 mm-thick and 0.025 mm-thin Cu wire aligned end-on or side-on to the beam, a 0.5 mm capillary of CeO_2_, and 0.5 mm Al wire.

Before describing the detailed modelling and experimental results, we highlight some key observations that motivate our approach. Fig. 2 ▸ shows the increase in temperature versus flux for all samples using temperatures calculated from the change in lattice parameter from X-ray diffraction.

A 0.5 mm capillary of CeO_2_ powder shows large increases in temperature under high flux, up to 210 K at 1.75 × 10^13^ photons s^−1^, with even small increases possibly having a significant effect on calibration and leading to inaccuracies in results (*e.g.* 200 K in temperature gives a strain of 2.2 × 10^−3^). The change in temperature was calculated from the change in lattice parameter, with a thermal expansion coefficient of 11 × 10^−6^ K^−1^ (Sameshima *et al.*, 2002 ▸). As CeO_2_ has a low thermal conductivity and was in powder form, it is difficult to model the thermal effects. Imaging of the CeO_2_ powder before and after exposure to the focused beam showed movement of the particles, further adding to the difficulty of modelling such a system. Consequently, having experimental data for such a system is paramount, and the results here show that beam heating may be significant.

A Cu wire sample with diameter 0.19 mm and length 0.85 mm showed the largest beam heating effect when oriented parallel to the beam, increasing by over 330 K. This beam heating was enough to cause recrystallization of the drawn wire sample, seen by the diffraction pattern changing from powder rings to intense spots in Fig. 5(*a*) later in the paper. This ‘thick’ Cu wire shows a non-linear relation between incident flux and temperature increase due to radiative cooling. The non-linear thermal expansion was accounted for in the calculation of temperature from the lattice parameter, as shown in Table 1 ▸ (Hahn, 1970 ▸).

Cu wire of the same length but 0.025 mm diameter showed a smaller temperature increase of 44 K. The average photon flux on this ‘thin’ wire is much higher than for the thick Cu wire as it is at the centre of the beam, with the beam size larger than the wire diameter. The lower temperature increases seen for the thin wire show that there are other factors, such as surface area to volume ratio, that influence beam heating effects.

For both thin and thick Cu wire samples, temperature increases are lower when side-on to the beam rather than end-on. As the same samples were used for both side-on and end-on measurements, only the path length of the beam, and therefore the absorbed power, will be different in the two scenarios, showing the significance of absorbed power on beam heating effects.

## 4. Testing the lumped thermodynamic model   

### 4.1. Model   

In order to better understand these beam heating effects for samples and experimental set-ups that may be expected in a typical materials science synchrotron experiment, we introduce a simple beam heating model. By experimentally testing this model, we can investigate the severity of the problem, and test a method of predicting such effects.

Kuzay *et al.* provide a clear analysis of beam heating effects, distinguishing between a ‘lumped’ model, where internal thermal conduction in the sample is negligible, and a ‘distributed’ model, where it is not (Kuzay *et al.*, 2001 ▸). As the focus of this study is on beam heating effects, rather than heat conduction models, we use the simpler lumped model.

For the lumped model to be a reasonable approximation, the thermal conductivity of the sample must be high enough or the sample size small enough that temperature is uniform across the sample on the time scales to be investigated [see Kuzay *et al.* (2001 ▸) for a quantitative description]. Consequently, beam heating effects should be dependent on radiation dose rate, *i.e.* the incident power, *P*, on the sample, rather than the total dose. Similarly, beam heating effects should be dependent on total incident flux and not flux density. This contrasts with macromolecular crystallography, where total dose is significant for both beam heating and radiation damage effects (Owen *et al.*, 2006 ▸; Sliz *et al.*, 2003 ▸; Leiros *et al.*, 2006 ▸).

The power absorbed by the sample, *P*_in_, is determined by the beam energy, *E*_b_, and flux, Φ, multiplied by the proportion of the beam absorbed by the sample: 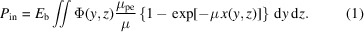
The portion of the total beam power, *P* = Φ*E*_b_, absorbed by the sample is calculated from the attenuation, μ, along the beam path through the sample, *x*, at a point *y, z* in the sample, multiplied by the flux at point *y, z* and integrated over all *y* and *z*. Previous works in the 10–15 keV range have used the total attenuation coefficient, recognizing that this is likely to be an overestimate (Rosenthal *et al.*, 2014 ▸; Wallander & Wallentin, 2017 ▸). At 10–15 keV, the generation of photoelectrons is the dominant mechanism of X-ray attenuation; however, at the higher energy used in this study, the contribution from Compton scattering is significant. Consequently, we can also consider the photoelectron contribution to the attenuation coefficient, determined by including the factor μ_pe_/μ, where μ_pe_ is the photoelectron attenuation coefficient, with values obtained from the XCOM database (Berger *et al.*, 2010 ▸).

For a sample larger than the beam size, and equal path lengths through the sample for all points in the beam (such as a wire aligned with its length along the beam), we can simplify equation (1) to 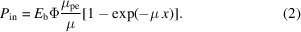
With a lumped model system, we can calculate the equilibrium temperature, *T*_eqm_, at which the rate of thermal energy deposition in the sample by the beam (*P*_in_) is equal to the rate of thermal energy loss (Maruyama & Moriya, 2021 ▸). With an environment temperature of *T*_0_, we find that 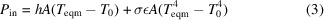
where the right-hand terms show the convective and radiative heat loss terms, respectively. The convective heat loss rate in equation (3) is dependent on surface area, *A*, and the heat transfer coefficient, *h*, while the radiative heat loss rate is dependent on surface area, emissivity, ε, and the Stefan–Boltzmann constant, σ.

Using a fourth-generation synchrotron source and the set-up typically expected for a material science experiment, it is possible to reach temperatures at which radiative effects are not negligible and a radiative cooling term should be included. This is shown by the relationship between Δ*T* = *T*_eqm_ − *T*_0_ and incident flux for the 0.19 mm-thick Cu wire shown in Fig. 2 ▸. Assuming *h* and ε to be independent of temperature, fitting this data with equation (3) gives an emissivity value of ε = 0.015 ± 0.004, matching literature values (Window & Harding, 1981 ▸; Estalote & Ramanathan, 1977 ▸).

Though solving equation (3) is straightforward, determining the appropriate value of *h* is not. Consequently, this has been a focal point for research on beam heating effects (Kriminski *et al.*, 2003 ▸). This heat transfer coefficient will depend on sample material, surface roughness, shape, orientation, and the surrounding temperature and airflow, making it challenging to determine.

By integrating equation (3), neglecting the radiative cooling term, the sample temperature at a time *t* after the X-ray beam is incident on the sample is found to be 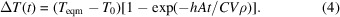
The characteristic time (*hA*/*CV*ρ) is dependent on surface area, *A*, volume, *V*, specific heat, *C*, and density, ρ (Kuzay *et al.*, 2001 ▸). This equation can be used to analyse time-resolved temperature measurements, allowing *h* to be experimentally determined for a specific sample and experimental set-up.

We can also consider the instantaneous sample heating that occurs before heat loss mechanisms become significant, *i.e.* adiabatic heating. In this case, the heating rate will be 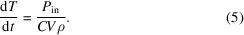
This simple model can be used to predict the changes in temperature for samples and experimental set-ups that may be expected in a materials science synchrotron experiment. By investigating the time dependence of temperature changes during beam heating, we will be able to determine the heat transfer coefficient for each sample. This value, along with the flux dependence of temperature changes, can then be used to calculate the thermal output of the system. Comparing this with the attenuated beam power will then show the effective ‘efficiency’ of beam heating.

We focus on using this model to predict scenarios with the highest increase in temperature in order to investigate the severity of the problem. Additionally, larger changes in temperature will allow for a more precise comparison with the model and improved sensitivity to the effects of radiative heat losses.

### 4.2. Aluminium wire results   

**Figure 3:**
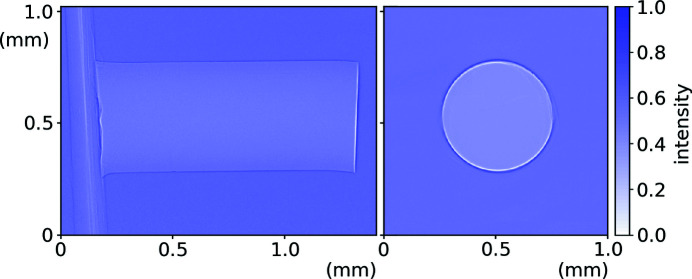
Side- and end-on radiographs of a 0.5 mm-diameter Al wire sample showing the Kapton support.

**Figure 4:**
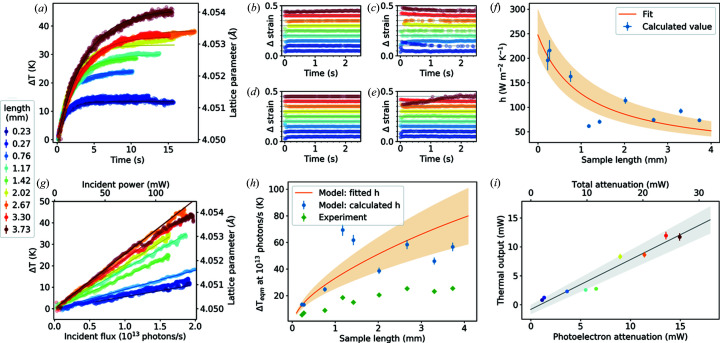
Results for 0.5 mm-diameter Al wire samples of varying lengths (see legend), with (*a*) the change in temperature as a function of time after insertion of focusing lenses into the beam, (*b*)–(*e*) change in strain of the (113), (222), (133) and (024) reflections, respectively, after insertion of focusing lenses, (*f*) calculated *h* values as a function of length, (*g*) change in temperature as a function of incident flux, (*h*) measured and modelled change in temperature at 10^13^ photons s^−1^, and (*i*) calculated thermal output at 10^13^ photons s^−1^ against X-ray beam attenuation.

A range of lengths of 0.5 mm-diameter Al wire were used to test the beam heating model with a low attenuation coefficient of μ = 1.2897 cm^−1^ and μ_pe_ = 0.7258 cm^−1^ (Berger *et al.*, 2010 ▸). Radiographs used to determine sample dimensions and align each sample to the beam are shown in Fig. 3 ▸. Fig. 4 ▸(*a*) shows the change in temperature of these wire samples as a function of time exposed to the focused X-ray beam. Temperature values were determined from the change in lattice parameter using a linear thermal expansion coefficient of 22 × 10^−6^ K^−1^ (Wilson, 1942 ▸).

Upon exposure to the focused X-ray beam, the initial rate of temperature increase (*i.e.* adiabatic heating rate) is the same for all Al wire lengths, showing that X-ray attenuation is approximately linear over the range of wire lengths measured. Because these measurements required fast acquisition of data, flux measurements could not be taken simultaneously and the values of Δ*T* could not be normalized and are therefore not comparable. Consequently, the heating efficiency cannot be calculated from adiabatic heating rate here.

As the Al wire samples have a diameter greater than the beam size, it is possible that there are internal thermal gradients. An examination of the change in strain calculated from each of the measured Bragg reflections gives further insight into this possibility. The values plotted in Figs. 4 ▸(*b*) to 4 ▸(*e*) show the difference between measured strain at a time *t* and strain at the final equilibrium temperature, *T*_eqm_, calculated from the (113), (222), (331) and (024) reflections, respectively, with the values for each length offset for clarity. Assuming the strain induced in the Al samples upon exposure to the focused beam is caused by thermal expansion, these strain values give an indication of the change in thermal gradient across the sample.

The longest samples measured clearly showed some changes, with the (222) reflections showing the greatest changes in strain over time for most samples. These changes occur over a time of under 2 s for the longest samples, and shorter times for shorter samples, showing that thermal equilibrium within the wire is reached quickly. As the changes in strain are faster than the time taken to reach thermal equilibrium, it is likely that the thermal impedance of the wire surface is much greater than that of the bulk of the wire. Consequently, we assume that the radial thermal gradient in the wire is likely to be negligible and so equation (4) should be a good approximation.

Each data set in Fig. 4 ▸(*a*) has been fitted with the function described in equation (4), shown with a solid line. When fitting, only values of the heat transfer coefficient, *h*, were allowed to vary. Values of *C* and ρ (see Table 1 ▸) and surface area and volume were kept constant with geometry calculated from radiographs, *e.g.* Fig. 3 ▸, assuming the wires to be cylindrical. All wire lengths show a good fit to this function, with the longer wire lengths clearly showing greater characteristic times. The results plotted in Fig. 4 ▸(*f*) show a decrease in *h* with length, fitted with an *h* ∝ 1/length trend.

Measured changes in temperature with increasing incident flux are plotted in Fig. 4 ▸(*g*), fitted with a linear trend, showing that radiative cooling was not significant. Some discrepancies can be seen, such as the dip seen for the 3.73 mm sample after 1.2 × 10^13^ photons s^−1^, which we believe to be due to movement of the sample. In such cases, only the data before the discrepancy were used for analysis.

The results from Fig. 4 ▸(*g*) are compared with the modelled temperature increase at 10^13^ photons s^−1^ in Fig. 4 ▸(*h*). These values were calculated by solving equation (3) for *T*_eqm_, using the values given in Table 1 ▸ and the calculated *h* values (blue circles), and the fitted *h* (length) function (orange line). Experimental values of *T*_eqm_ (green diamonds) show a similar trend to the modelled values with calculated and fitted *h* values, but with experimental values lower than modelled. Experimental values are found to be around 50% of the predicted temperature increases, with the exception of values for the 1.17 and 1.42 mm wires, which show a larger discrepancy. We find that *T*_eqm_ increases with length as absorption is approximately linear over this length range.

The calculated thermal output at equilibrium calculated for an incident flux of 10^13^ photons s^−1^ (69.6 mW at 43.44 keV) is plotted against attenuated power calculated from the photoelectric absorption coefficient in Fig. 4 ▸(*i*). The fitted gradient of 86 ± 9% shows the effective heating efficiency relative to the photoelectric attenuation. If only the total attenuation coefficient is used, a lower total heating efficiency of 48 ± 5% is obtained. The 1.0 ± 0.9 mW *x* intercept shows that photoelectron losses are not significant on the length scales being considered here.

### 4.3. Copper wire results   

**Figure 5:**
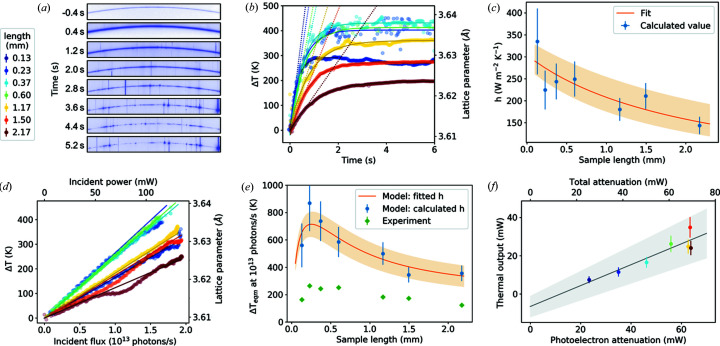
Results for 0.19 mm-diameter Cu wire samples of varying lengths (see legend), with (*a*) images of the (222) reflection as a function of time after insertion of focusing lenses into the beam from a 0.86 mm-length sample, (*b*) the change in temperature as a function of time after insertion of focusing lenses, (*c*) calculated *h* values as a function of length, (*d*) change in temperature as a function of incident flux, (*e*) measured and modelled change in temperature at 10^13^ photons s^−1^, and (*f*) calculated thermal output at 10^13^ photons s^−1^ against X-ray beam attenuation.

Similar measurements and analysis were performed on 0.19 mm-diameter Cu wire samples with a range of lengths, with results shown in Fig. 5 ▸. Cu has μ = 34.6 cm^−1^ and μ_pe_ = 31.8 cm^−1^ at 43.44 keV, greater than the values for Al, and the wire cross section is comparable with the beam size, leading to significant beam heating effects. This is displayed in Fig. 5 ▸(*a*), where the diffraction rings from a 0.86 mm-length sample are shown for a range of times, with focusing lenses inserted into the beam at 0 s. The powder rings initially increase in intensity and shift to lower **Q** before separating into intense spots around the ring, showing the heating and subsequent recrystallization of the drawn wire.

Fig. 5 ▸(*b*) shows the change in temperature as a function of time exposed to the focused X-ray beam. Temperature transients of these Cu samples occur over less than 2 s, faster and to much higher temperatures than the Al samples. As fast flux measurements could not be taken simultaneously, the magnitude of these temperature changes cannot be normalized and compared. Fits to equation (4) are shown with a solid line, while the adiabatic heating rate is shown with a dotted line. The noise in the data for samples that reached higher temperatures is due to high-intensity reflections appearing as a result of recrystallization. This noise impeded strain analysis; however, as the wire diameter size is close to the beam size and attenuation through the sample length is approximately linear, it is reasonable to assume that internal conduction did not have a significant effect on characteristic time.

The rapid beam heating caused some movement of the samples, as seen in Fig. 5 ▸(*d*) for the two longest samples. Linear fits were used to analyse these data, taking the steepest gradient for each data set, which removes contributions from sample movement and radiative cooling. With these gradients it is possible to compare the measured temperature changes with the model at 10^13^ photons s^−1^, as shown in Fig. 5 ▸(*e*). The data and model using calculated and fitted *h* values have similar shape, with a peak in *T*_eqm_ at around 0.25 mm length. However, there are large differences between the model and experiment, with predicted values increasing over 860 K, while experimental values do not go above 265 K. The discrepancy between model and experiment is evident when the thermal output is plotted against the calculated photoelectric attenuation in Fig. 5 ▸(*f*). Fitted with a linear trend, the large errors from the calculated *h* values and surface area give an *x* intercept of 12 ± 10 mW. The fitted gradient gives a heating efficiency of 55 ± 10% relative to the calculated photoelectric attenuation and 50 ± 10% relative to the calculated total attenuation.

### 4.4. Comparison of Al and Cu   

Comparing the results from the 0.19 mm Cu and 0.50 mm Al wires, we find some differences, both with their fit to the lumped model and measured efficiency. The heating rate in the adiabatic regime (before heat loss becomes significant) is the same for all Al wires but decreases with increasing wire length for Cu due to the high attenuation of X-rays in Cu. This difference in attenuation between Al and Cu is also evident when comparing Fig. 4 ▸(*h*) and Fig. 5 ▸(*e*), where the experimental and modelled temperature changes decrease after only 0.25 mm length, while for Al they continue to increase over the measured range of sample lengths.

Al and Cu wires also showed differences in the measured photoelectron heating efficiency, with values of 86 ± 9% and 55 ± 10%, respectively. These differences and the values themselves may be understood by considering unaccounted energy loss mechanisms. Heat lost through the Kapton holder may contribute to energy losses. The effect of wire length on this conductive cooling would be more significant for shorter wire lengths, which is the pattern seen for Cu samples, and may explain the non-zero *x* intercept seen in Fig. 5 ▸(*f*). We would expect the rate of heat lost through conduction to be proportional to the temperature difference Δ*T*, which correlates with the lower thermal efficiency and greater *x* intercept seen for the Cu samples compared with Al. Additionally, we expect the rate of heat lost through conduction to be proportional to the contact area between the Kapton and the wire, *i.e.* the wire cross section. Heat loss through the Kapton holder should be more significant for the thicker Al wire, but this does not seem to be the case. Perhaps the temperature gradient from the Al wire to the Kapton is not large enough to drive heat loss via conduction.

In our model, we have only used the photoelectron cross section of the material under investigation to determine the deposited energy, and have not considered the various energy loss mechanisms of the photoelectrons, not all of which will be converted to thermal energy. Electron escape is expected to be on a scale much smaller than our samples, with photoelectrons spreading on the order of 200 nm from the absorption event (Torsello *et al.*, 2018 ▸; Nave & Hill, 2005 ▸), meaning that a much higher level of precision would be required to see such effects here. It is challenging to compute photoelectron energy loss mechanisms such as fluorescence and Auger electron generation; however, we can make some simple assessments. The fluorescence yield is dominated by *K*α emission for both Al and Cu (Krause *et al.*, 1978 ▸). For Al the *K*α energy is 1.5 keV with an attenuation length of 0.009 mm, so very few fluorescence photons will escape the 0.5 mm-diameter Al wire. Cu *K*α emission at 8.0 keV has an attenuation length of 0.022 mm which is about 10% of the wire thickness. Fluorescence photons emitted from the centre of the wire will be reabsorbed but those emitted from nearer the surface may escape. Energy loss via fluorescence emission may partially explain the difference in the measured photoelectron heating efficiency between Al and Cu samples.

Bonino *et al.* used a more complex theoretical model using Monte Carlo (MC) simulations considering both photo-atomic and electron interactions to calculate the proportion of the incident X-ray beam converted into thermal energy in the sample (Bonino *et al.*, 2020 ▸). Comparing explicit (using MC) to implicit modelling (using Beer–Lambert) of the experimental results of Snell *et al.* (2007 ▸), Bonino *et al.* find around 1 K difference between explicit and implicit models for a 5 to 10 K increase in the temperature of a glass bead in a 15 keV X-ray beam. This gives a heating efficiency similar to that seen for the Al wire samples. Though their experimental and modelled values are similar, the significant differences in their time dependence show the limits of the model, making it difficult to draw any conclusions about the thermal efficiency and therefore the accuracy of the model using MC simulations.

## 5. Discussion   

Our results show significant beam heating effects for materials science samples in a monochromatic hard X-ray beam. Using high-energy X-rays allows the use of transmission geometry and bulk measurements, favouring the use of samples such as capillaries of powders, plates, wires or small single crystals. Radiation damage effects have not previously been a concern for these radiation-resistant materials, but we have seen that beam heating effects can become significant for certain sample sizes even at high X-ray energy with high flux. Consequently, when performing synchrotron experiments, beam heating effects should be considered, especially when using a high-flux focused beam and absorbing samples which match the beam size.

Knowing the potential effect of beam heating, we can now consider how to predict and mitigate these effects. Predicting the exact temperature increases due to beam heating can be challenging, but by considering samples as thermally isolated and assuming maximum heating efficiency, we can use a lumped model to give an upper limit of temperature increase. In this case, the main challenge is determining *h*, which will be dependent on sample shape, orientation and environment. There are many formulas for calculating *h* using the Nusselt, Grasof and Prandtl dimensionless parameters, but few seem suited to small samples. [Calculated values for the samples used here based on the work of Incropera *et al.* (2007 ▸) gave *h* values around 50 to 200 Wm^−2^ K^−1^ lower than the experimentally determined values.] Consequently, calculated values of *h* may not give accurate predictions, but may be useful as a conservative low estimate of *h* for getting an upper limit of predicted temperature increase. In low-pressure or vacuum environments, samples will be better thermally isolated and *h* decreases significantly so that beam heating is a much more severe problem.

An alternative to predicting beam heating effects is to check if they are present during an experiment by measuring with reduced beam flux. Results may be compared to quickly see evidence of beam heating effects.

Beam heating effects may be reduced in several ways. Using equation (3), we can see that heating is reduced by increasing the ratio of the surface area to length along the beam. Where possible, it may help to maximize the heat transfer coefficient, *h*, through sample orientation and exterior cooling. Providing thermal connection to a heat sink is also likely to be an effective way to increase the heat loss rate, reducing sample heating. If a sample has a thermal connection to a heat sink with a length *l*, thermal conductivity *k* and cross-sectional area *D*, assuming the convective cooling of the heat sink connection is negligible, a further term can be added to the equation for equilibrium temperature, giving 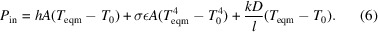
Beam properties will also have significant effects on beam heating. We have shown that at a given energy *T*_eqm_ is proportional to photon flux when radiative cooling is not significant. Beam heating effects can be reduced by lowering flux; however, this will slow down measurements and prevent fast processes from being studied.

We may also increase the speed of measurements, in order to complete them before *T*_eqm_ is reached. In the instantaneous heating regime, we expect adiabatic heating with Δ*T* proportional to total dose before the heat loss becomes significant. This diffraction before destruction approach has been extensively discussed for macromolecular crystallography. The ultimate realization is perhaps serial crystallography, where the sample is completely destroyed by the X-ray beam.

The fast measurement approach will be most effective for samples with larger characteristic times, *i.e.* samples with low thermal conductivity or thermally isolated samples *e.g.* in vacuum. For thermal insulators, heat is not as effectively dissipated through the sample, and therefore decreasing the flux density will also decrease beam heating effects, *e.g.* using an unfocused beam rather than a focused beam when measuring a CeO_2_ calibration sample and spending a few seconds longer to collect the data.

## 6. Conclusions   

The high flux levels achievable at fourth-generation synchrotron sources offer many advantages for measuring weak signals; however, it is important to be aware of possible effects of these high radiation levels. We have shown that X-ray beams can cause significant thermal, physical and structural changes to metal samples, clearly demonstrating that the effects of beam heating are of potential concern for a wide range of samples and can have a profound impact on experimental results. Consequently, it is vital to be able to understand and predict these effects.

By performing measurements on a system designed to be realistic as an experimental set-up but to simplify modelling of the system, we have been able to test a model for beam heating predictions. Comparison between model and experiment showed these predictions are accurate at determining the relative effects of attenuation coefficient and sample size. However, discrepancies were found between the results of Al and Cu wire samples, with Cu samples showing lower heating efficiency. This discrepancy may be because far higher temperatures are reached by Cu samples, or other unaccounted heat loss mechanisms. A better understanding of energy loss mechanisms will be needed to accurately predict the magnitude of temperature changes. Overall, these predictions provide an experimentally verified starting point for analysis of more complex samples and experiments, and have given useful insight into how to predict and mitigate beam heating effects.

## Figures and Tables

**Table 1:** Values used for calculation of experimental results Density, ρ, is calculated using experimental lattice parameters.

	Thermal expansion (×10^−6^ K^−1^)	μ (cm^−1^)	μ_e_ (cm^−1^)	*C* (J g^−1^ K^−1^)	ρ (g cm^−3^)
Al	22 (Wilson, 1942 ▸)	1.2897 (Berger *et al.*, 2010 ▸)	0.7258 (Berger *et al.*, 2010 ▸)	0.9 (Leadbetter, 1968 ▸)	2.68
Cu	11.5 + 2.43 × 10^−2^ *T*	34.5856 (Berger *et al.*, 2010 ▸)	31.763 (Berger *et al.*, 2010 ▸)	0.39 (White & Collocott, 1984 ▸)	8.95
	−2.88 × 10^−5^ *T* ^2^ + 1.47 × 10^−8^ *T* ^3^ (Hahn, 1970 ▸)				
CeO_2_	11 (Sameshima *et al.*, 2002 ▸)				
